# Social capital, perceived stress, and mental health of men who have sex with men in China: A cross-sectional study

**DOI:** 10.3389/fpsyg.2023.1134198

**Published:** 2023-03-30

**Authors:** Xiaoyue Zhang, Ying Zhou, Kaili Zhang

**Affiliations:** School of Nursing, Xuzhou Medical University, Xuzhou, China

**Keywords:** men who have sex with men, social capital, social network, social participation, perceived stress, mental health

## Abstract

**Background:**

Mental health problems are prevalent among men who have sex with men (MSM). Social capital and perceived stress may affect the mental health of MSM. The purpose of this study was to understand the current status of mental health, social capital, and perceived stress among MSM and to explore which variables are influential factors of mental health.

**Methods:**

This study adopted a convenience sampling method and posted recruitment information through online social platforms (Blued, QQ, and WeChat) from January 2022 to June 2022 to recruit participants. The questionnaire included a demographic questionnaire, Social Capital Questionnaire (SCQ), Perceived Stress Scale (PSS), and Self-Reporting Questionnaire 20 (SRQ-20). Descriptive analyses of demographic characteristics, social capital, perceived stress, and mental health were conducted using percentages, median, interquartile range, means, and standard deviations. *One-way ANOVA* and independent-samples *t-test* were used to test the relationship between demographic characteristics and mental health, and multiple linear regression was used to analyze which variables were influencing factors of mental health. *SPSS 24.0* was used for data analysis, and significant differences were found at *p* < 0.05.

**Results:**

A total of 546 MSM participated in this study. Total social capital score was 18.59 ± 2.62, cognitive social capital, social participation, and social network scores were 6.53 ± 1.05, 4.15 ± 0.97, and 7.91 ± 1.57. PSS score was 26.91 ± 6.44, and SRQ-20 score was 7.5 (3, 11). Education level, relationship status, employed information, monthly income, sexual orientation, perceived stress, and three dimensions of social capital were included in the multiple linear regression based on the results of *One-way ANOVA*, *t*-test, and correlation analysis. Multiple linear regression analysis showed that relationship status (in a relationship), sexual orientation (heterosexual, bisexual, other), perceived stress, social participation, and social network had a significant effect on mental health.

**Conclusion:**

Sex with men have poorer mental health. Relationship status, sexual orientation, perceived stress, social networks, and social participation are important factors influencing mental health. The general public should be called upon to treat them with a more tolerant attitude, improve the social environment, and promote their identification with their sexual orientation, thereby reducing perceived stress and promoting the mental health of this population. In addition, from the perspective of social capital, promoting MSM social participation and expanding social networks may also be an important way to promote MSM mental health.

## 1. Introduction

Men who have sex with men (MSM), including gay, bisexual men, men who identify as transsexual, and men who have sex with men who are heterosexual. Compared to the general population, MSM in China are more likely to suffer from mental health problems, including depression, anxiety, suicidal behavior, and substance dependence (Yu et al., 2013; Sun et al., 2020). A meta-analysis on the mental health of MSM from China showed that 43.2% of participants reported depressive symptoms, 32.2% of participants MSM reported anxiety symptoms, 21.2% had suicidal ideation, 6.2% had suicide plans, and 7.3% had attempted suicide (Wei et al., 2020). In addition, men who have sex with men are also at high risk for HIV infection. The prevalence of depressive symptoms among HIV-positive MSM in China was 44.1% (Zhang et al., 2018). The reasons of mental health problems among MSM are various and may be related to stress and stigma caused by social exclusion (Reisner et al., 2016), self-identity (Zhang et al., 2014), high-risk sexual behavior (Fendrich et al., 2013), and other factors. Despite the bleak state of mental health among MSM, only a small percentage of MSM actively seek and receive appropriate health services.

The contemporary term social capital became popular in the late 1980s with the research of Pierre Bourdieu and James Coleman. In 2000, Putnam proposed the importance of social capital for health outcomes (Rodgers et al., 2019). When the World Health Organization developed a new European policy for health—Health 2020, one of the prominent factors that was identified as a protective and promoting factor was “social capital” (World Health Organization, 2013). Social capital embraces the supportive resources inherent in an individual’s social network and his or her capacity to mobilize them (Yang and Yang et al., 2010; Wu D. et al., 2021). In general, the multidimensional structure of social capital can be divided into cognitive social capital and structural social capital. Cognitive social capital refers to subjective self-assessment of social relationships, such as social trust and perceived reciprocity among neighbors. Structural social capital is assessed through objective indicators such as participation in social organizations, volunteering, social participation, and civic activities (McKenzie et al., 2002). This study focuses on social capital at the individual level, and the assessment of individual social capital consists of cognitive social capital, the social networks that individuals have, and participation in social activities (Lynch et al., 2000; Brune and Bossert, 2009).

Related research has found that social capital interventions can be effective in improving subjective well-being, social support, resilience, sense of community, and quality of life, and reducing loneliness and depression (Flores et al., 2018). A study in Iran showed a significant relationship between mental health and social capital in the workplace; the higher the social capital of workers, the lower the risk of psychological distress, and enhancing social capital can be considered as a means of improving employees’ mental health (Firouzbakht et al., 2018). A Japanese study showed that all three factors of social capital (trust, connection and interaction, and social participation) and happiness were positively correlated (Tsuruta et al., 2019). Although the relationship between social capital and mental health has been well established in other populations, there are still relatively few studies on social capital and mental health in MSM. A study on the relationship between suicidal ideation, stigma, and social capital among MSM in three West African countries showed that social capital can reduce suicidal ideation among MSM (Stahlman et al., 2016). Therefore, social capital may be an important factor influencing mental health in MSM.

In China, influenced by Confucianism, collectivism, and a traditional culture that promotes heterosexual marriage, procreation, and filial piety, sexual minorities may suffer prejudice, stigma, and discrimination because of their sexual orientation (Fu et al., 2020; Yan et al., 2020). A study suggests that the high prevalence of mental health problems such as depression, anxiety, suicidal behavior, and alcohol abuse among Chinese MSM can be explained by minority stress (Sun et al., 2020). MSM are also more likely to suffer from depression during the process of minority stress (Meyer, 2003). A study of gay-related stressful events and emotional distress in MSM from China showed that over a 12-month period, participants had at least three to four times the prevalence of suicidal ideation, suicide planning, and suicide attempts than adult males in the general Chinese population. And an increase in gay-related stressful events significantly increased the risk of suicidal ideation and suicide planning among participants (Yu et al., 2018). Stress refers to the process of adaptation once a person is exposed to external or internal challenges (Chrousos, 2009). Maladjustment to stress not only alters brain function and peripheral physiology and can lead to a range of psychological and organic disorders. Perceived stress can be seen as a result of the interaction between the individual and the environment, is a cognitive evaluation process of the events acting on the individual (Katsarou et al., 2012). There are few studies on the status of perceived stress among men who have sex with men, but perceived stress has been found to be potentially related to mental health in other populations. A study of perceived stress and emotional distress showed a significant positive correlation between perceived stress and emotional distress (Yan et al., 2021). Also, perceived stress had a significant positive predictive effect on depression (Sebena et al., 2012). During the COVID-19 epidemic, higher perceived stress was associated with more emotional distress, including depression, fear, obsessive–compulsive anxiety, neurosis, and hypochondria (She et al., 2021). Based on previous studies, this study suggest that perceived stress may be an important factor affecting the mental health of MSM，and effective regulation and control of perceived stress can reduce the generation of negative emotions, such as anxiety and depression in MSM, and prevent the impact on the mental health of individuals.

In addition, Meyer’s (Meyer, 2003) minority stress model explains the impact of stress on sexual minorities from three aspects: general stressors, distal minority stress, and proximal minority stress. Various factors in the environment can lead to stress (general stress). Stress due to minority status (including sex orientation, race/ethnicity, and gender) is distal minority stress (e.g., prejudice, discrimination, and violence). Stress due to one’s own minority identity is minority proximal stress (e.g., expectations of rejection, concealment, and internalized homophobia). All three aspects of stress can have an impact on mental health.

In conclusions, mental health problems in MSM are persistent and severe, but most research on MSM has focused on HIV infection prevention and intervention, and researchers have often overlooked the importance of mental health. At present, social capital is relatively rare in the study of mental health of MSM. Based on previous research and minority stress models, this study selected appropriate questionnaires and scales to investigate the demographic characteristics, social capital, perceived stress, and mental health status of MSM, and analyzed the influencing factors of the mental health of MSM, so as to provide references for strengthening the psychological intervention and promoting the mental health status of MSM.

## 2. Methods

### 2.1. Study population and sample size

This study adopts convenience sampling method and snowball sampling. Inclusion criteria were: (1) 18 years of age or older; (2) biological sex was male and reported having had sex (including oral and anal sex) with another male within the past 12 months; and (3) no serious medical conditions, able to communicate normally, read and write, and consent to participate in this study. Exclusion criteria were: those with a dyslexia or mental deficiency or those who were unable to understand survey questions. The formula as follows:


n=Zα22P1−PDδ2

*P* is the overall proportion of a characteristic, and the maximum sample size to meet the survey demand is obtained when *P* is taken as 0.5. *D* is the design efficiency and takes values between 1.5 and 2.5. *δ*^2^ is the precision and takes values between 0.01 and 0.25. The effective return rate of the survey questionnaire is calculated according to 80%. The maximum sample size for this study was approximately 300.

### 2.2. Data collection

This study posted recruitment information through online social platforms (Blued, QQ, and WeChat) from January 2022 to June 2022 to recruit eligible participants. Before filling out the questionnaire, we obtained the informed consent of the respondents. In addition, the study was completed anonymously and participants were assured that the survey data would be used only for this study to protect them from the risk of privacy exposure. In this study, the questionnaires were mainly filled out online. To ensure the authenticity of the respondents, firstly, the researcher would control that a cell phone could only fill out the questionnaire once through the backend of the questionnaire filling software to avoid the questionnaire being filled out repeatedly. Then, the snowball sampling method was used to recruit participants by adding friends or joining same-sex dating group chats on QQ or WeChat; Blued, a large domestic gay male dating service platform, mainly recruited participants by posting dynamics or sending one-on-one chat messages. Finally, once participants completed their questionnaires, researchers would review the completeness and logic of the questionnaire in the backstage. If the questionnaire is approved, the subjects will get a red envelope of 10 yuan as compensation for their time. This study was approved by the Ethics Committee of the Xuzhou Medical University.

### 2.3. Measurement tools

#### 2.3.1. Demographics questionnaire

The demographic questionnaire included age, residence (city and countryside), education level (technical secondary school/high school and below, junior college, undergraduate, and graduate), marital status (single, in relationship, and divorce/widowed), working condition (employed and unemployed/retired/student), monthly income, sex orientation (homosexuality, heterosexuality, bisexuality, and other), and sex role (top, bottom, and versatile). In this study, the other options for sexual orientation were MSM who were transgender, pansexuality, or still unsure of their sexual orientation.

#### 2.3.2. Self-reporting questionnaire 20

The Self-Reporting Questionnaire 20 (SRQ-20) is a simple and rapid screening tool for mental disorders issued by the World Health Organization (WHO; Beusenberg and Orley, 1994), with a total of 20 items. Each entry is rated “0” or “1.” 1 indicates that these symptoms have existed in the past month, while 0 indicates that the symptoms do not exist. A score of ≥7 indicates the participant need psychological assistance due to emotional distress, but no specific symptoms have been identified. A score of >15 indicates that the participant has poor mental health and need for psychological treatment or psychiatric treatment. The higher the score, the worse the patient’s mental health. The *Cronbach’α* coefficient of this scale in this study was 0.868.

#### 2.3.3. Social capital questionnaires

Social Capital Questionnaires (SCQ), compiled by Yang et al. (2010), are used to survey social capital at the individual level. There are 12 items in the SCQ. Wu et al. (2016) divided the questionnaire into three dimensions. The aspects pertained to cognitive social capital based on responses (dichotomized as yes/no) to four questions (e.g., “You believe that most people around you will help you when needed”), three questions on social participation (e.g., “How many times did you participate in leisure or entertainment activities organized by your school or community during the past year?”), and five questions addressing social network (assessing number of good friends, trusted classmates, helpful neighbors, close relatives, and cooperative partners). The higher the score, the richer the social capital. The *Cronbach’α* coefficient of this questionnaire in this study was 0.650.

#### 2.3.4. Perceived stress scale

The Perceived Stress Scale (PSS), developed by Cohen et al. (1983), is used to measure people’s ability to feel excessive pressure on their situations in life. Yang and Huang translated it into Chinese Perceived Stress Scale (CPSS; Yang and Huang, 2003), with 14 entries. It consists of seven negative and seven positive items, each with a possible answer rated on a five-point scale from 0 (never) to 4 (very often). The highest possible score is 56. Items 4, 5, 6, 7, 9, 10, and 13 are the positive items, positive items are scored in reverse，and were rated from 4 to 0. The range of scores was between 0 and 56. A score less than 28 means low perceived stress, and a score equal to or greater than 28 means high perceived stress. The higher the total score, the more stress the individual felt. The *Cronbach’α* coefficient of this scale in this study was 0.734.

### 2.4. Data analysis

This study used *Epi Data 3.0* software (The Epi Data Association, Odense, Denmark) to entry data. Moreover, all descriptive and inferential statistics were conducted using *SPSS 24.0* (SPSS Inc., Chicago, IL, United States). Descriptive analyses of demographic characteristics, social capital, perceived stress, and mental health were conducted using percentages, median, interquartile range, means, and standard deviations (SD). *One-way ANOVA* and independent-samples *t-test* were used to test the relationship between demographic characteristics and mental health. *Spearman’s* correlation analysis was used to examine the correlation between social capital, perceived stress, and mental health. Multiple linear regression was used to analyze which variables were factors influencing the mental health of MSM. The dependent variable was mental health, and the independent variables were demographic characteristics (education level, relationship status, employed information, monthly income, and sexual orientation), perceived stress, and social capital (cognitive social capital, social participation, and social network). *p* < 0.05 was considered statistically significant.

## 3. Results

### 3.1. General information and comparison of mental health

A total of 624 questionnaires were collected in this study, of which 546 were valid. The age of the participants ranged from 18 to 49, with an average age of 27.77 ± 7.77. The participants’ education level was mostly undergraduate (58.2%), their relationship status was mostly single (11.0%), their work status was mostly employed (63.2%), their sexual orientation was mostly homosexual (50.7%) or bisexual (29.3%), and with 65 participants (11.9%) identifying themselves as heterosexual. The sample distribution is detailed in Table 1.

**Table 1:** Effects of demographic characteristics on mental health (*n* = 546).

Variable	*N* (%)	Mental health (Mean ± SD)	*t*/F	*p*
Age			0.153	0.858
18–28	318 (58.2)	7.56 ± 5.38		
29–39	175 (32.1)	7.41 ± 5.04		
≥40	53 (9.7)	7.17 ± 3.87		
Residence			−1.661	0.097
City	348 (63.7)	7.20 ± 5.10		
Countryside	198 (36.3)	7.95 ± 5.19		
Education level			8.265	<0.001
Technical secondary school/High school or below	68 (12.5)	9.07 ± 4.81		
Junior college	145 (26.6)	8.65 ± 5.04		
Undergraduate	288 (52.7)	6.53 ± 5.16		
Postgraduate	45 (8.2)	7.31 ± 4.54		
Relationship status			3.411	0.034
Single	341 (62.5)	7.86 ± 5.34		
In relationships	172 (31.5)	6.63 ± 4.66		
Divorce/Widowed	33 (6.0)	7.94 ± 5.03		
Employed information			−2.049	0.041
Employed	345 (63.2)	7.13 ± 5.11		
Unemployed / Retired / Student	201 (36.8)	8.06 ± 5.14		
Monthly income (CNY)			3.929	0.020
<3,000	181 (33.2)	7.93 ± 5.31		
3,000–5,000	197 (36.1)	7.76 ± 4.91		
>5,000	168 (30.8)	6.67 ± 5.14		
Sex orientation			3.953	<0.001
Homosexual	277 (50.7)	6.78 ± 5.16		
Heterosexual	65 (11.9)	7.52 ± 5.13		
Bisexual	160 (29.3)	8.41 ± 5.06		
Other	44 (8.1)	8.39 ± 4.78		
Sex role			0.613	0.542
Top	173 (31.7)	7.64 ± 5.37		
Bottoms	191 (35.0)	7.65 ± 5.02		
Versatile	182 (33.3)	7.13 ± 5.04		

### 3.2. Self-reporting questionnaire 20 score

The SRQ-20 scores in this study ranged from 0 to 20, and the median and interquartile range were 7.5 (3, 11). In this study, 48.9% of the participants scored between 7 and 15, and 7.1% of them scored >15. The higher SRQ-20 scores of participants suggested that MSM had poorer mental health status. The results of the independent-samples *t-test* and *one-way ANOVA* revealed statistically significant differences in the scores of education level, relationship status, employed information, monthly income, and sexual orientation in relation to mental health among the demographic characteristics. See Table 1 for details.

### 3.3. Social capital questionnaires score

The Social Capital Questionnaire scores for men who have sex with men ranged from 13 to 24 points, with a score of the SCQ was 18.59 ± 2.62. The cognitive social capital score was 6.53 ± 1.05, and the score for each item in this dimension was 1.63 ± 0.26. The social participation score was 4.15 ± 0.97, and the score for each item in this dimension was 1.38 ± 0.32. The social network score was 7.91 ± 1.57, and the score each item in this dimension was 1.58 ± 0.31.

### 3.4. Perceived pressure scale score

The score of the Perceived Stress Scale for MSM was 26.91 ± 6.44, with a score of 1.92 ± 0.46 for each item. 46.1% of the participants scored ≥28 on the PSS, representing their high level of perceived stress.

### 3.5. Correlation analysis between social capital, perceived stress, and mental health among MSM

*Spearman’s* correlations between variables are shown in Table 3. The results showed that individuals with higher levels of social capital, including cognitive social capital, social participation, and social network, had lower SRQ-20 scores, representing better mental health. Individuals with higher levels of perceived stress also had higher SRQ-20 scores, representing poorer mental health.

**Table 3:** Correlations analysis of social capital, perceived stress, and mental health (*n* = 546).

Variables	1	2	3	4	5	6
Social capital	—					
Cognitive social capital	0.629^***^	—				
Social participation	0.612^***^	0.121^***^	—			
Social network	0.858^***^	0.313^***^	0.350^***^	—		
Perceived pressure	−0.340^***^	−0.221^***^	−0.193^***^	−0.287^***^	—	
Mental Health	−0.292^***^	−0.134^***^	−0.166^***^	−0.305^***^	0.512^***^	—

^*^p < 0 0.05, ^**^p < 0 0.01, and ^***^p < 0.001.

### 3.6. Factors associated with mental health

To explore which variables are influencing factors to mental health, multiple linear regression analysis was used. This study defined mental health as the dependent variable, and variables such as education level, relationship status, employed information, monthly income, sex orientation, perceived stress, and social capital were included in the multiple linear regression analysis according to Tables 1, 3. Before the analysis, the variables involved were assigned, among which the unordered multicategorical variables, including relationship status (set dummy variable with reference to “single”) and sex orientation (set dummy variable with reference to “homosexuality”), needed to set dummy variables.

According to Table 2, variables such as relationship status (in relationships), sex orientation (heterosexual, bisexual, and other), perceived pressure, social participation, and social network had a significant effect on mental health. The largest effect was on perceived stress (β = 0.523, *p* < 0.001), followed by social networks in social capital (β = −0.158, *p* < 0.001).

**Table 2:** Factors associated with MSM mental health based on multiple linear regression analysis (*n* = 546).

Variables	Nonstandard coefficient		Standard coefficient (β)	*t*	*p*	95%CI
	*B*	S.E.				
Education level	−0.412	0.226	−0.065	−1.822	0.069	−0.856 ~ 0.032
Relationship status						
In relationships	−1.125	0.436	−0.102	−2.579	0.010	−1.982 ~ −0.268
Divorce/widowed	0.266	0.785	0.012	0.339	0.735	−1.276 ~ 1.807
Employed information	−0.501	0.492	−0.047	−1.081	0.309	−1.467 ~ 0.456
Monthly income	0.442	0.309	0.069	1.429	0.154	−0.166 ~ 1.050
Sex orientation						
Heterosexuality	2.075	0.611	0.131	3.399	0.001	0.876 ~ 3.275
Bisexuality	1.023	0.420	0.091	2.436	0.015	0.198 ~ 1.848
Other	1.646	0.686	0.087	2.400	0.017	0.298 ~ 2.993
Perceived pressure	0.417	0.030	0.523	13.750	<0.001	0.358 ~ 0.477
Cognitive social capital	0.042	0.200	0.008	−0.209	0.835	−0.350 ~ 0.434
Social participation	−0.352	0.186	−0.072	−1.892	0.049	−0.118 ~ −0.013
Social network	−0.519	0.131	−0.158	−3.953	<0.001	−0.777 ~ −0.261

*R*^2^ = 0.366, *F* = 25.675, and *p* < 0.001.

## 4. Discussion

In this study, 48.9% of the participants scored between 7 and 15, and 7.1% of them scored >15, indicating poor mental health in this group. Similar to the results of previous studies, a systematic review from China showed that the combined prevalence of depressive or depressive symptoms among MSM was 40.0%, much higher than the reported incidence in the general Chinese male population (0.54–2.2%; Fu et al., 2020). The summary prevalence of anxiety symptoms among all MSM was 12.2–57.6% (Wei et al., 2020). The causes of serious mental health problems among MSM are multiple. In terms of population size, China has the largest homosexual population in the world (Wang et al., 2019), there is an estimated 21 million MSM in the sexually active phase (Wu W. et al., 2021). And on a social level, the status of sexual minorities may make it more difficult for them to seek medical or social help. In addition, influenced by traditional Chinese family values, most sexual minorities may be excluded from their families and lack support from their loved ones and families, which may also lead to poorer mental health status of MSM (Hu, 2016; Wei et al., 2020). Therefore, mental health issues in MSM require special attention.

This study found that relationship status (in relationships) was an important factor in mental health, indicating that MSM in a stable relationship had better mental health than single MSM. Similar to previous studies, a study on the effects of relationship quality and loving attitudes on self-stigma and mental health in HIV-positive MSM showed that MSM who had emotionally involved committed relationships or disclosed their HIV-positive status to their intimate partners had better happiness than other HIV-positive MSM (Yang et al., 2017). It follows that high-quality relationships and positive attitudes toward love provide another source of positive self-perception and well-being (Kim and Hatfield, 2004; Yang et al., 2017). A variety of behaviors in intimate relationships, including emotional contact, companionship, and physical intimacy, can provide feelings of attachment, trust, and satisfaction (Fletcher et al., 2000). A study from the United States and Poland showed that individuals in relationships reported better health, especially if they were in a high-quality relationship (Adamczyk et al., 2021). A stable, high-quality relationship may provide higher levels of psychosocial support, economic resources, health, and self-esteem for each other, leading to higher life satisfaction and coping styles that buffer the effects of adverse life events and promote mental health (Fagan, 2009; Siva Kumar et al., 2019). When intimate relationship is lacking, young people may be at risk for health problems. Although having a stable relationship may be beneficial to the development of MSM’s mental health, most of the MSM in this study were single, and the reason for this situation may be related to the lack of positive self-perception and self-stigma (Yang et al., 2017). There are not many studies on MSM relationship status and mental health, and the next research should focus on exploring what factors influence MSM relationship status and relationship quality, so as to create good conditions for MSM to find a stable and high-quality relationship.

In addition to relationship status, this study found that men who identified themselves as heterosexual, bisexual, or other sexual orientation had poorer mental health compared to homosexual. MSM’s cognition of their sexual orientation is also an important factor in mental health. Men’s mental health status may be influenced by their internal gender identity (Wei et al., 2020). Similar to previous studies, MSM had a high negative self-identification rate of 75.8%, with the majority of MSM not identifying themselves as homosexual (Zhang et al., 2014). A study conducted in urban western and southwestern China showed a 50.9% prevalence of depression among men who have sex with men and women, and 35.2% among men who have sex with men (Hu et al., 2019). Similarly, findings from other countries also suggest that bisexual men face greater mental health problems than homosexual men (Conron et al., 2010; Kipke et al., 2020). The reasons for this discrepancy in China are not yet clear, but studies in other Western countries suggest that poor mental health among bisexuals may be related to bisexual invisibility, erasure, bisexual-specific experiences of discrimination, biphobia in the homosexual community, and lack of support for bisexuality (Ross et al., 2018). It follows that improving MSM’s identification with their sexual orientation can also be a way to promote mental health. Therefore, the public should be called upon to treat MSM with more tolerance, especially the bisexual group among MSM or those with lower sexual orientation identity, and to improve and reduce the bi-phobia and discrimination in the surroundings of MSM. It is also important to strengthen support for MSM from society, community, friends, and family to promote their identification with their sexual orientation and thus improve their mental health.

Social capital to be a significant contributor to mental health outcomes (Bassett and Spencer, 2013; Ehsan and De Silva, 2015). This study found that social capital was positively correlated with mental health, and that social participation and social network were important factors influencing mental health. A study of social capital and suicidal ideation among MSM in West African countries found that participation in social activities, such as church, clubs, or broader community activities, was significantly associated with a reduction in suicidal ideation (Stahlman et al., 2016). Several studies have identified a correlation between social participation, volunteerism, and mental health (Thoits and Hewitt, 2001; Diener and Seligman, 2002). And social isolation can have a negative impact on mental health, especially for stigmatized groups such as MSM (Lackner et al., 1993; Liu and Mustanski, 2012). Many studies have demonstrated that the participation dimension of social capital is associated with mental health status, but not many studies have been conducted on social networks, since only a few disclosed that the network dimension of social capital was related to depressive symptoms (Bassett and Spencer, 2013). A study on uncertainty stress, social capital, and suicidal ideation among Chinese medical students found that participants with smaller social networks were more likely to experience suicidal ideation (Wu W. et al., 2021). In MSM, one study found that in the group of men who have sex with men, coming out without social support or a narrow social network will result in the adverse effect of stress, depression, and suicidal ideation and attempts (Cho and Sohn, 2016).

Therefore, intervention from the perspective of social capital, especially social participation and social networks, may be one of the effective ways to improve the mental health of MSM. First of all, relevant institutions can formulate policies to encourage the development of voluntary organizations and communities of sexual minorities and create a more harmonious environment for these organizations to develop. Secondly, these voluntary organizations or communities can organize more health education and communication activities according to their own situation to provide more social opportunities for MSM. Finally, through publicity and education, gay men can be encouraged to actively Finally, through publicity and education, MSM can be encouraged to participate in activities organized by schools, workplace, voluntary organizations, or communities to enhance social participation, broaden the social circle of MSM, and expand their own social networks, so as to improve the mental health level of MSM. In addition, MSM can positively influence health behaviors by spreading health-related information to each other through the social networks they have, establishing healthy social norms, monitoring and preventing unhealthy behaviors, and fostering personal responsibility for health (Thoits and Hewitt, 2001).

The cognitive component includes perceptions of support, trust, reciprocity, and shared values. But in this study, the results showed that cognitive social capital has no significant effect on mental health among MSM, which might be attributed to: Firstly, in recent years, with the opening and modernization of Chinese society, homosexuality has become increasingly visible in China, but an overall negative perception of MSM currently remains in China (Hu, 2016; Fu et al., 2020). Secondly, communities and non-governmental organizations about sexual minorities are very few in China. Although they hope to find more people who are similar to themselves and share a common language through these organizations, and they are eager to receive more support and care from these organizations. However, because these organizations are not well developed, they are unable to provide enough services to meet their needs for physical or mental health services (Xie et al., 2019). Social capital plays an important role in the health of MSM, further research is needed to discover the specific role of social capital in the mental health of MSM, and it is also important to understand the mechanisms by which social capital affects the mental health of MSM.

In this study 46.1% of participants scored >28 on the PSS, indicating a higher level of perceived stress among MSM. Also, perceived stress is an important factor affecting mental health. A study from Korea showed that the prevalence of perceived stress and depression among MSM was 46.7 and 42.7%, and the perception of the self as a stigmatized and devalued MSM correlated with perceived stress and suicidal ideation (Cho and Sohn, 2016). And a study conducted among sexual minorities found that worse self-assessment of health status was associated with higher perceived stress (Michalski et al., 2021). The reason for the higher perceived stress in MSM may be that MSM experience discrimination, stigma, rejection, and prejudice in their lives because of their sexual orientation. The accumulation and interaction of these pressure sources and the lack of social support can make stress management difficult and lead to mental health problems (Yu et al., 2018). Therefore, a harmonious and friendly environment should be created to reduce the prejudice of others against MSM and to reduce the pressure source in daily life, thus reducing the stress and promoting the mental health of MSM.

## 5. Limitation

(1) This study is a cross-sectional study and causal inferences cannot be made. (2) Men who have sex with men are hidden, and the questionnaire was only distributed and collected online in this study. This leads to the possibility that the sample in this study is not well representative of overall MSM, and the generalizability of its findings is limited. (3) There are many variables that affect the mental health of MSM, but how each variable plays a role in influencing mental health is not clear at present. The next step can focus on the study of the mechanism of influence and discuss the specific relationship between each variable through path analysis.

## 6. Conclusion

This study found that relationship status, sexual orientation, perceived stress, and social capital all have important effects on the mental health of MSM. This suggests that it is still important to first call on the general public to treat MSM with a more tolerant attitude, improve the overall social environment, reduce external discrimination and prejudice against MSM, and promote the identification of MSM’s sexual orientation, while reducing the generation of external and internal pressure source and relieving MSM’s perceived stress. Secondly, the development of voluntary organizations and communities for sexual minority should be encouraged, through which more health education and services are provided to MSM to make them feel more belonging. Meanwhile, these organizations or communities can provide more opportunities for MSM to participate in activities, thus expanding the social participation and social network of MSM and improving the level of social capital. Finally, it is important to focus on the mental health problems of MSM, to provide mental health services and interventions for MSM with more targeted and effective approaches according to the different situations of individuals, and to encourage MSM to actively seek mental health services, which is also one of the effective ways to improve the mental health of MSM.

## Data availability statement

The raw data supporting the conclusions of this article will be made available by the authors, without undue reservation.

## Ethics statement

The studies involving human participants were reviewed and approved by Ethics Committee of the Xuzhou Medical University. The patients/participants provided their written informed consent to participate in this study.

## Author contributions

All authors listed have made a substantial, direct, and intellectual contribution to the work and approved it for publication.

## Funding

This study was supported by Excellent Talents Research Foundation of Xuzhou Medical University (grant number D202026).

## Conflict of interest

The authors declare that the research was conducted in the absence of any commercial or financial relationships that could be construed as a potential conflict of interest.

## Publisher’s note

All claims expressed in this article are solely those of the authors and do not necessarily represent those of their affiliated organizations, or those of the publisher, the editors and the reviewers. Any product that may be evaluated in this article, or claim that may be made by its manufacturer, is not guaranteed or endorsed by the publisher.
