# Supplementary material for: Social capital, perceived stress, and mental health of men who have sex with men in China: A cross-sectional study
Source: Front Psychol. 2023 Mar 30;14:1134198. doi: 10.3389/fpsyg.2023.1134198 (PMC10098019; doi:10.3389/fpsyg.2023.1134198)
Supplement: Supplementary file 1 [file Data_Sheet_1.pdf]

Supplementary Material

Social Capital, Perceived Stress and Mental Health of Men who have Sex with Men in China: A Cross-sectional Study

1 The demographic questionnaire

(1) What is your age ? ____

(2) Where is your present residence?

①City; ②Countryside

(3) What is your current education level?

①Technical secondary school / High school and below; ②Junior college; ③Undergraduate; ④Graduate

(4) What is your current relationship status?

①Single; ②In relationship; ③Divorce / Widowed

(5) What is your current employed information?

①Employed;②Unemployed / Retired / Student

(6) What is your current monthly income (CNY)?

①<3000;②3000 ~5000;③>5000

(7) What is your sexual orientation?

①Homosexuality (You think you can only be emotionally, lovingly or sexually attracted to the same sex);

②Heterosexuality (Although you have had sex with someone of the same sex, you still believe you are only emotionally and sexually attracted to the opposite sex);

③Bisexuality (You think you can be emotionally, lovingly or sexually attracted to both the same and opposite sex);

④Other (You are transgender, pansexual, or still unsure of your sexual orientation)

(8) What is your sexual role when you have sex with men?

①Top; ②Bottoms; ③Versatile

2 Supplementary Table

Table 1 Factors associated with Mental Health among MSM and their assignment

Variables	Assignment
Education level	Technical secondary school / High school and below=1, Junior college=2, Undergraduate=3, Graduate=4
Relationship status	Single:(x_1=0,x_2=0)
	In relationship:(x_1=1,x_2=0)
	Divorce / Widowed:(x_1=0,x_2=1)
Employed information	Employed=0, Unemployed / Retired / Student=1
Monthly income	＜3000=1, 3000-5000=2, ＞5000=3
Sex orientation	Homosexuality:(x_1=0,x_2=0,x_3=0)
	Heterosexuality:(x_1=1,x_2=0,x_3=0)
	Bisexuality:(x_1=0,x_2=1,x_3=0)
	Other:(x_1=0,x_2=0,x_3=1)
Social capital	
Cognitive social capital	Continuous variable
Social participation	Continuous variable
Social network	Continuous variable
Perceived pressure	Continuous variable
**Dependent Variables**	
Mental health	SRQ-20 score
